# Supplementary material for: Doxepin Exacerbates Renal Damage, Glucose Intolerance, Nonalcoholic Fatty Liver Disease, and Urinary Chromium Loss in Obese Mice
Source: Pharmaceuticals (Basel). 2021 Mar 16;14(3):267. doi: 10.3390/ph14030267 (PMC8001117; doi:10.3390/ph14030267)
Supplement: Supplementary file 1 [file pharmaceuticals-14-00267-s001.pdf]

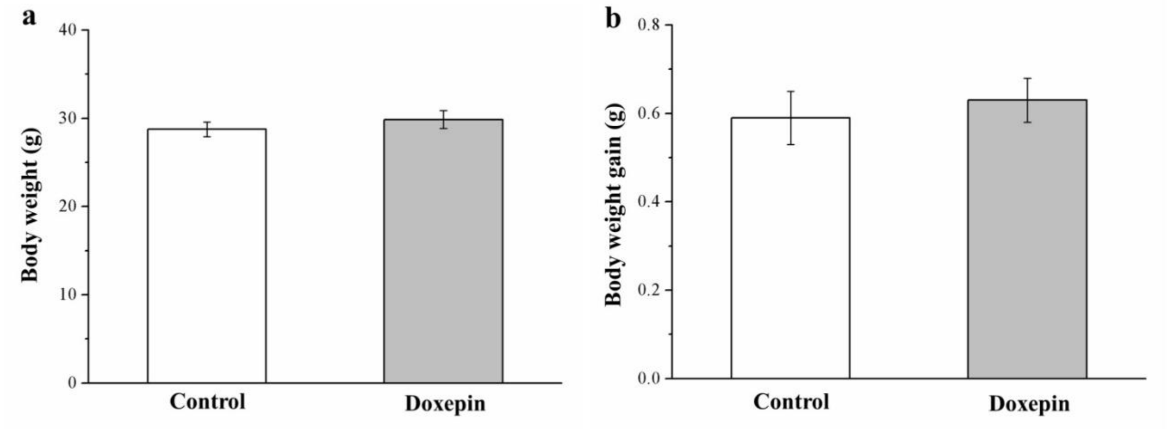

Figure S1. Body weight and body weight gain in controls and mice on an SD treated with doxepin (5 mg/kg/day) over 56 treatment days.

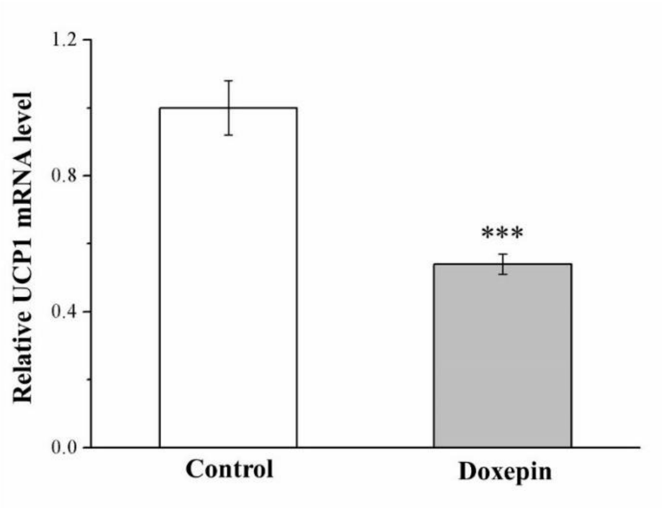

Figure S2. *UCP1* mRNA expression in the brown adipose tissue of control and doxepin-treated obese mice over 56 treatment days.

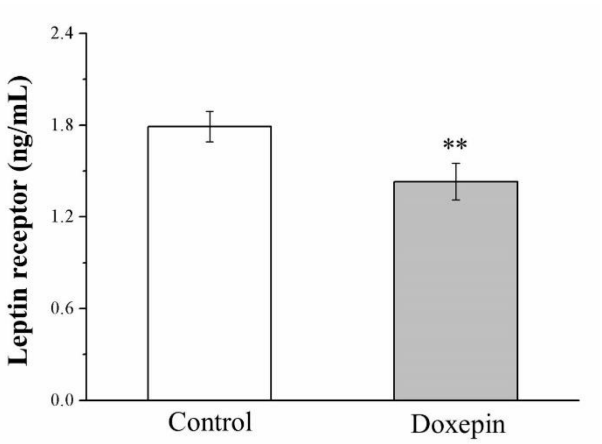

Figure S3. Serum-soluble leptin receptor levels of controls and doxepin-treated obese mice over 56 treatment days.

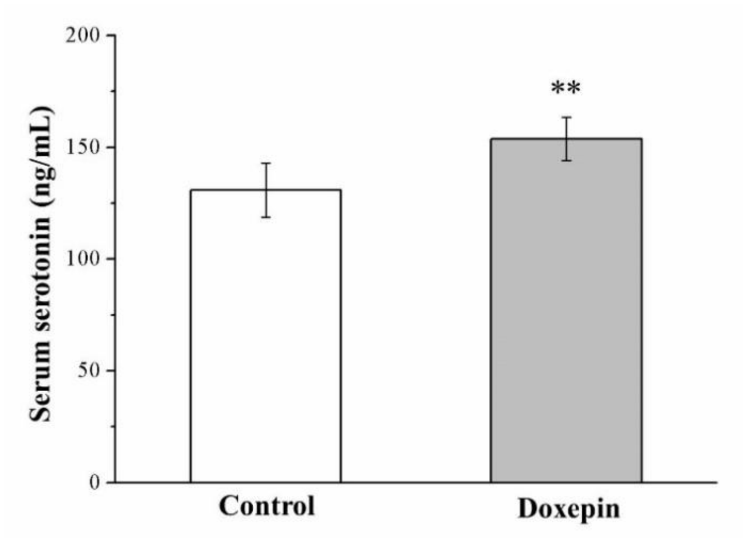

Figure S4. Serum serotonin levels of controls and doxepin-treated obese mice over 56 treatment days.

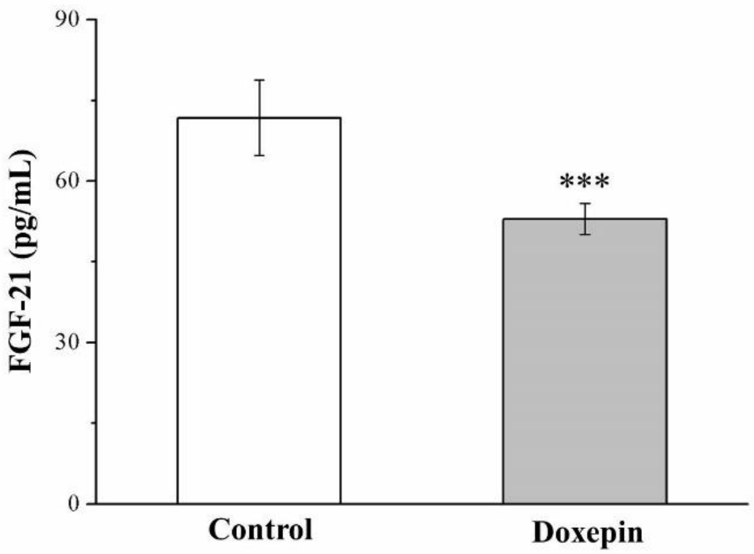

Figure S5. Serum FGF-21 levels of controls and doxepin-treated obese mice over 56 treatment days.

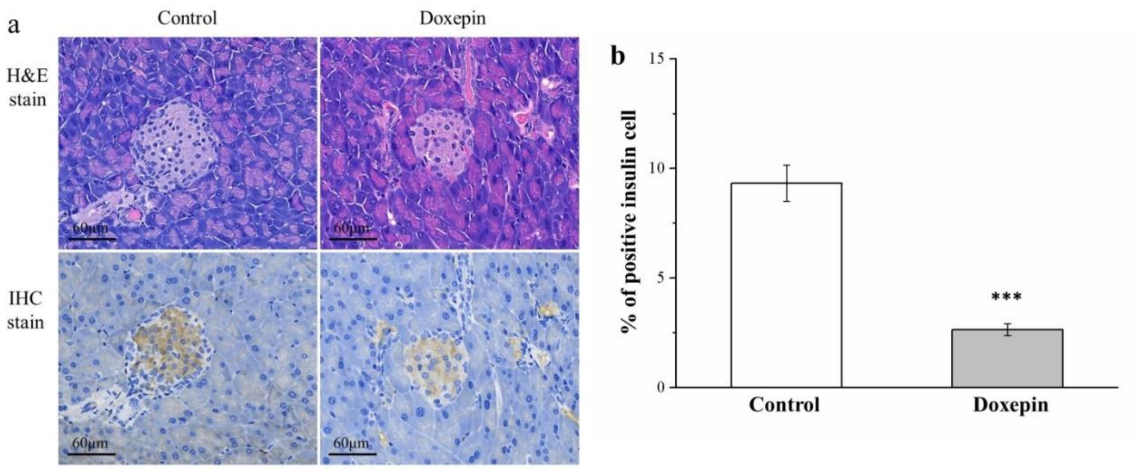

Figure S6. (**a**) Islet morphology (H&E staining and IHC) and (**b**) β-cell percentage of controls and doxepin-treated obese mice over 56 treatment days (magnification, 200×).

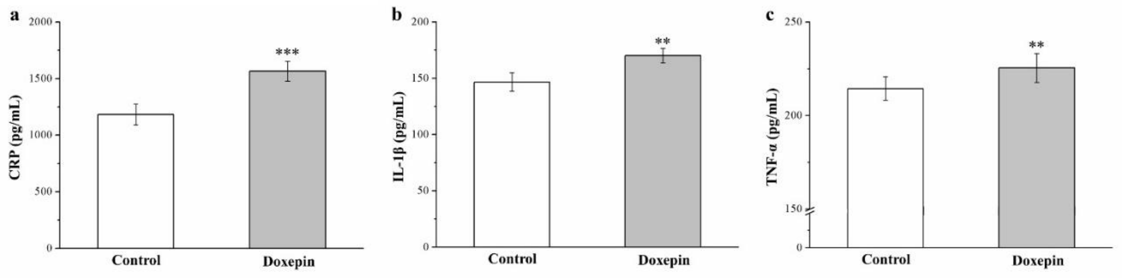

Figure S7. Serum (**a**) CRP, (**b**) IL-1β, and (**c**) TNF-α levels in controls and doxepin-treated obese mice over 56 treatment days.

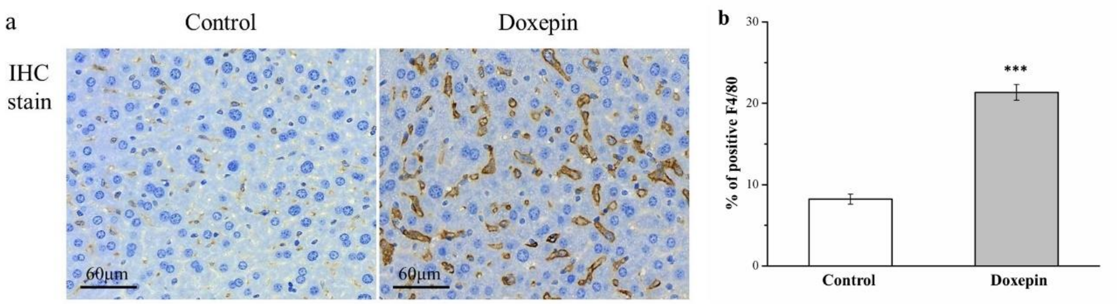

Figure S8. (a) IHC and (b) expression of F4/80 in the livers of controls and doxepin-treated obese mice over 56 treatment days (magnification, 200×).

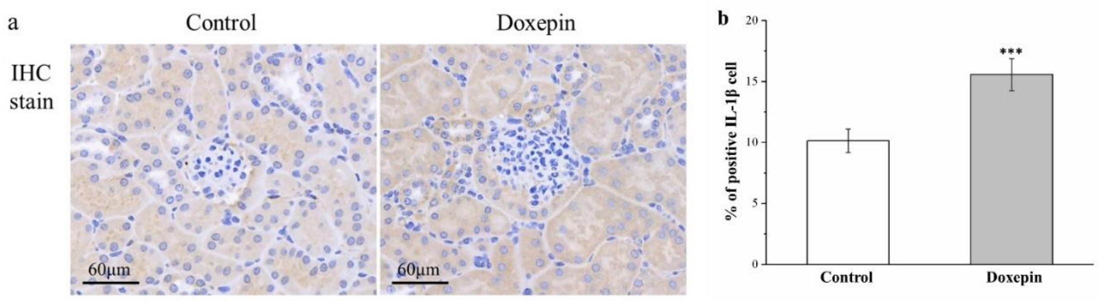

Figure S9. (**a**) IHC for and (**b**) expression of IL-1β in the kidneys of controls and doxepin-treated obese mice over 56 treatment days (magnification, 200×).

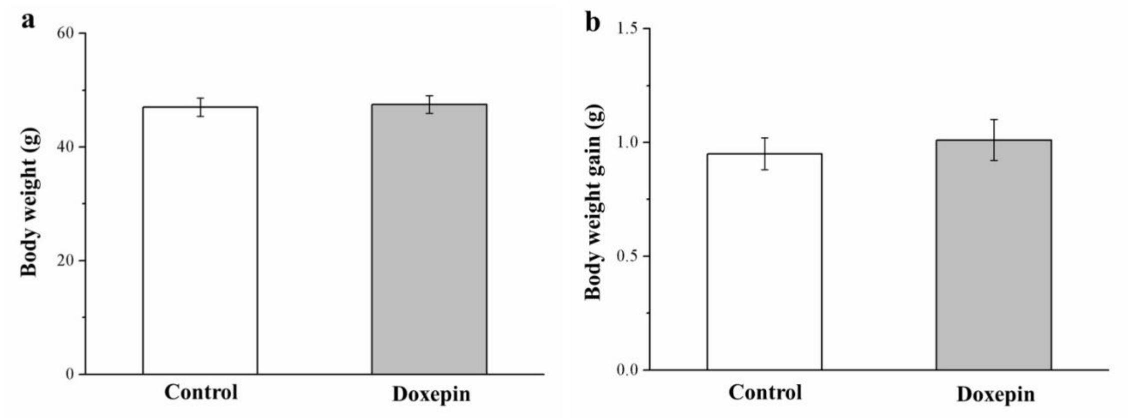

Figure S10. Body weight and weekly body weight gain in controls and mice receiving doxepin treatment (2 mg/kg/day) over 56 treatment days.
